# Electro Fluid Dynamics: A Route to Design Polymers and Composites for Biomedical and Bio-Sustainable Applications

**DOI:** 10.3390/polym14194249

**Published:** 2022-10-10

**Authors:** Nergis Zeynep Renkler, Iriczalli Cruz-Maya, Irene Bonadies, Vincenzo Guarino

**Affiliations:** Institute of Polymers, Composites and Biomaterials, National research Council of Italy, Mostra d’Oltremare Pad.20, V. le J.F. Kennedy 54, 80125 Naples, Italy

**Keywords:** electrospinning, electrospraying, nanofibers, microgels, nanoparticles, biomaterials

## Abstract

In the last two decades, several processes have been explored for the development of micro and/or nanostructured substrates by sagely physically and/or chemically manipulating polymer materials. These processes have to be designed to overcome some of the limitations of the traditional ones in terms of feasibility, reproducibility, and sustainability. Herein, the primary aim of this work is to focus on the enormous potential of using a high voltage electric field to manipulate polymers from synthetic and/or natural sources for the fabrication of different devices based on elementary units, i.e., fibers or particles, with different characteristic sizes—from micro to nanoscale. Firstly, basic principles and working mechanisms will be introduced in order to correlate the effect of selected process parameters (i.e., an applied voltage) on the dimensional features of the structures. Secondly, a comprehensive overview of the recent trends and potential uses of these processes will be proposed for different biomedical and bio-sustainable application areas.

## 1. Introduction

Nowadays, a wide range of biomaterials—from various synthetic or natural sources—are continuously processed with an ever-increasing control of the structural features in order to create 3D platforms suitable for tissue engineering applications and various therapeutic applications. Over the relevance of constituent materials and their physical/chemical properties, it is well known that peculiar structural features at different scales can affect a wide range of final product properties. For instance, several studies remarked that nano or microparticles could be more advantageous for injection applications than those with bigger sizes ranging from hundred microns up to millimeters [1]. Likewise, in the case of fibrous scaffolds used in vitro, it is known that nano-textured surfaces, i.e., fiber diameter at the nanoscale, are able to better influence the behavior of living cells in terms of biomechanical, biological (i.e., cell adhesion and proliferation) and fluid/molecular transport properties rather than micro-surfaces, thus mimicking the typical architecture of basement lamina membrane, present in all the natural tissues [2]. Lastly, for bio-filtering applications, the role of mesh size in nanofibrous membranes is a determinant to improving separation efficiency, thus meaning a better capacity to remove bio-contaminants in medical solutions, higher flux in separation, and lower energy for operation [3]. Therefore, it is crucial to employ the processing techniques available to preserve control of their structural properties at micro- and nano-levels to impart, case by case, morphological and topographical cues tailored for the specific application [4]. In the last decade, various cost-effective and reproducible manufacturing techniques have been developed to produce micro and nanostructured materials with the desired characteristic sizes. In the literature, different methods were described to produce micro-and nanofibers, including interfacial polymerization, self-assembly, and freeze-drying [5]. Among them, Electro Fluid Dynamic processes (EFDs) currently identify one of the most versatile and cost-effective class of manufacturing technologies for the manipulation of polymers or composites, generally from melt or solution, by the application of high voltage electric field, to produce fibers or particles from micro to nanoscale [6,7]. They include two different working modes, electrospinning and electrospraying, that allow producing, respectively, non-woven fibers or particles of different sizes and shapes. They are highly tunable in their characteristic size and shape by accurately selecting the materials/solvents/additives and finely controlling the process parameters [8]. Moreover, many materials, such as synthetic and natural polymers, metals, ceramics, composites, and blend systems can be sagely combined for the fabrication of multicomponent devices suitable in many fields such as tissue engineering and regenerative medicine [9], drug delivery [10], wound dressing [11], bioelectronics [12], and biofiltration systems [13].

Despite the recent goals in terms of research outcomes in the biomedical field, upscaling EFD technologies for clinical translation and large-scale production is still so far due to several problems concerning the current biomanufacturing processes, mainly related to the use of volatile solvents and all the risk factors connected to health and safety standards during fabrication and product implementation [14].

It is well known that the solvent evaporation mechanism drives conventional electrospinning. Environmental damage associated with using organic solvents with low boiling points—namely Trifluoroethanol (TFE) and dichloromethane (DCM)—may not be thoroughly recommended by the U.S. Food and Drug Administration for the fabrication of electrospun fibers for pharmaceutical applications. Moreover, solvent traces may be entrapped into the fiber body after resting treatments, thus restricting their use for biological use that requires direct contact with the cells [15].

In this review, an overview of the different EFD processes was proposed. In the first part, working principles were introduced, highlighting some recently explored insights to minimize the manufacturing impact on the environment (i.e., melt electrospinning, green electrospinning). In the second part, a wide range of blends or composite devices based on fibers or particles with different size scales were systematically discussed according to their application in the biomedical and environmental fields.

## 2. Electro Fluid Dynamics: Classification and Working Principles

In the last two decades, a large variety of technologies based on “bottom-up” or “top-down” manufacturing techniques was investigated, highlighting relevant benefits and some drawbacks. In contrast to the top-down approach, bottom-up methods first attempt to produce micro-scale structures that can be put together to develop complex structures. They are described as a two-stage fabrication process that includes the final assembly of these structures after they are created [16,17]. This bottom-up approach is essential to processing and manipulating small-scale biomaterials using specific technologies such as microfluidic techniques [18]. Among them, EFDs can be considered a class of “bottom-up” technologies that use high-voltage electric forces on viscoelastic polymers at the melt state or in solution form. It includes two main processing modes: electrically assisted spinning and spraying—suitable to synthesize micro- and nanostructures in the form of particles or fibers [6]. Their popularity is mainly due to the high reliability and versatility of these processes that allow fabricating a plethora of smart devices with controlled sizes from micro- to submicrometric scale and multiple functionalities as a function of the specific experimental configuration used.

**Figure 1:**
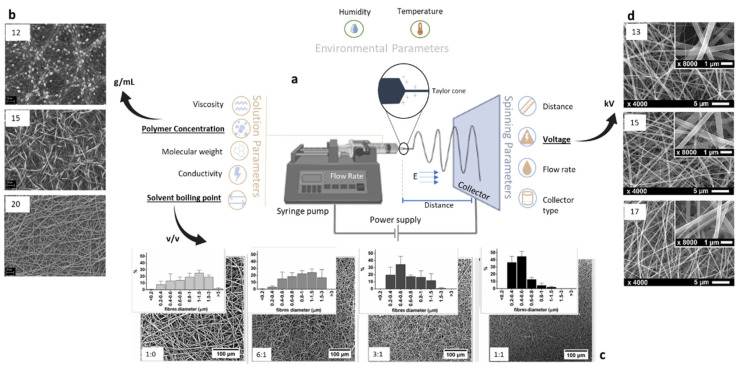
(**a**) Scheme of the basic electrospinning setup and influence of solution parameters: (**b**) Polymer concentration (SEM images of PCL fibers at different concentrations from [20]) and (**c**) solvent boiling point (SEM images and diameter size distribution of poly(trimethylene carbonate-co-ϵ-caprolactone) [P(TMC-CL)] nanofibers from [21]—spinning parameters: (**d**) applied voltage (SEM images of PVP/Cellulose Acetate/Garlic nanofibers(scale bar 5 μm-int 1 μm) from [22].

The most elementary configuration is straightforward and includes three components: a high-voltage power supply, a pump system where a syringe/cartridge is arranged, and a ground collector (Figure 1a). Polymer comes out of the needle tip at a constant rate with the support of pumping forces until a droplet is formed [19].

Two opposite forces contribute to forming the droplet: the electrostatic force acting on this droplet shape and the opposite force, the surface tension force, that tends to preserve the droplet’s spherical shape. At the point that the two forces have equilibrium, it is expressed by the following equation:

$$\frac{1}{8p\varepsilon o}.\frac{Q2}{R2}=8p\sigma sR$$

where *Q* is the charge located on the droplet surface, *R* is the droplet radius, ε_o_ is the vacuum permeability, and σ_s_ is the surface tension coefficient. The charge increases by increasing the strength of the electric field, and the value of this electrostatic field force can be controlled by varying the voltage at a fixed distance between the two electrodes [23]. Up to a critical point where the electrostatic force overcomes the surface tension, the droplet is then disrupted into smaller droplets in electrospraying [5]. The solution will become a conical droplet, a Taylor cone, when using a higher molecular weight polymer. Due to sufficient chain entanglement structure, hydrophobic interactions, and hydrogen bonds of the polymer lead to a stable, continuous fiber formation instead of droplets. When the electrostatic force overcomes the surface tension, it stretches the jet stream causing it to elongate as a filament. This filament solidifies and is eventually deposited on a grounded collector, these ultrafine fibers will form, and this process is then called electrospinning [24].

In the electrospinning process, many parameters determine spinnability and the final product’s morphology and uniformity of nanofibers. Different nanofibers with different morphologies can be fabricated by sagely dosing and combining these parameters in uncountable ways. Solution-dependent parameters (i.e., viscosity, polymer concentration, solvent type, surface tension, electrical conductivity, and molecular weight) (Figure 1b,c) and environmental conditions, such as humidity and temperature, directly affect spinnability. The set up parameters can be adjusted during processing. For instance, spinning distance, applied voltage, flow rate, and collector type also affect spinnability and the final product morphology [25,26]. It is known that the polymer concentration affects the fiber diameter from micro- to nano-size by changing the viscosity, and together with surface tension, it is directly proportional to fiber diameter. As the viscosity increases, the fiber diameters also increase. In addition, the fiber diameter can be affected by adjusting the distance, voltage, and flow rate during the process [27,28]. Especially the applied voltage is a critical factor because the formation of fibers only occurs when the applied voltage exceeds the threshold voltage [29]. Additionally, it directly affects fiber morphology, as an increase in applied voltage leads to an increase in the electrostatic force of the polymer solution and a decrease in fiber diameter with the stretching of the jet (Figure 1d) [22]. However, it should be noted that these parameters do not affect the fiber morphology alone; for example, the effect of voltage on the fiber diameter will vary according to the polymer solution concentration and the distance between the tip and the collector [30].

Numerous and varied polymers can be processed by electrospinning but often requires the use of toxic organic solvents. The awareness of the disadvantages associated with the use of toxic solvents has increased interest in using non-toxic (benign) solvents for electrospinning. However, many benign solvents are not directly suitable for electrospinning, and longer and more accurate process optimization is required to obtain fibers with suitable morphology [20]. For instance, it has been reported that PCL, a polymer frequently used in biomedical applications, applies to electrospinning using less harmful solvents, including formic acid/acetic acid. Moreover, Van der Schueren et al. showed that increasing the formic acid in the solution decreases the mean fiber diameter [31].

**Figure 2:**
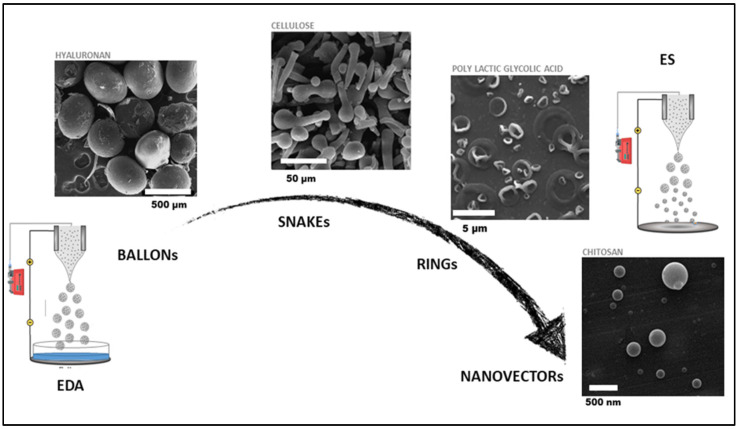
Fabrication of particles based on biomedical/bio-sustainable polymers from macro to nanoscale switching between EDA to ES mode. EDA process is mainly dedicated to the fabrication of balloons made of polysaccharides (i.e., chitosan, alginate) and/or glycosaminoglycans (i.e., hyaluronic Acid [39]) in water solution. EDS process is mainly devoted to the fabrication of nanovectors—i.e., nanoparticles with sub-micrometric or nanometric diameters—using highly volatile solvents (i.e., acetic acid, ethyl acetate). Specific modification of the experimental process setup can impart different sizes and shapes to final particles (i.e., snakes, Rings) [40].

EFDTs also represent useful tools of current materials science and processing to fabricate micro- and nanoparticles made of polymers from the natural or synthetic origin, overcoming some limitations of other consolidated technologies in this area, i.e., micro-emulsification, self-assembly, and solvent evaporation. They can be classified into two different groups: electrospraying (ES) and electro hydrodynamic atomization (EDA) (Figure 2) [32]. Both technologies are based on applying an electric field to nebulize a highly dilute polymer solution into droplets with highly controlled and uniform diameters. In particular, ES is a continuous process where the mechanism of polymer jet deformation and disruption into small particles is due to the Coulombic repulsion onto the surface of highly charged droplets against cohesive forces among polymer chains [33]. The solvent evaporation can be controlled by varying the distance between the tip-nozzle and collector to ensure the formation of spherical and solvent-free nanoparticles [34,35]. Contrariwise, EDA is a non-continuous mode, or dripping mode, where the gravity forces—due to the drop density and weight—and electric forces compete to overcome the surface tension at the needle tip. However, they are strongly counteracted by the peculiar properties of the used solvent (i.e., permittivity, boiling point) that drastically limit the capability of droplets to surface overcharge [36]. In this case, solvent removal can be obtained by collecting droplets into a bath via chemical or physical ways (i.e., gelation, solvent/non-solvent extraction) that allow freezing the particle sizes into a dimensional range from tens to hundreds of micrometers. The chance to minimize chemical additives, working at ambient temperature/humidity conditions, means that they are a sustainable choice to process biologically derived and biodegradable materials for different bio-inspired applications. Moreover, numerous versatility of the process setups, i.e., coaxial-ES/EDA, co-ES/EDA, and sequential ES/EDA, allows for a mix of different materials for the fabrication of multi-component systems that may combine particles and/or fibers for countless uses [37,38].

## 3. Melt Polymers vs. Polymer Solution

### 3.1. Fibers from Melt Polymers

Melt electrospinning is a simple method to produce polymeric fibers without using organic solvents. This environmentally friendly approach offers several advantages in fiber manufacturing, including avoiding the use of solvents to dissolve the polymers, as in the case of solution electrospinning.

The basic apparatus generally consists of a high-voltage power source, a needle, a heating device (heating oven, heat guns, laser melting devices, and electric heating), a melted polymer, and a collector. Similar to polymer solution electrospinning, larger diameter nanofibers are obtained using polymers with higher molecular weight in the melt electrospinning system. The electrospun melt mats generally consist of fibers both on the nanometer and micrometer scales [5]. Basically, in a melt electrospinning system, the melt polymer forms a cone at the tip of the capillary when the voltage is applied. After a critical voltage, electrostatic forces cause the surface tension of the melt polymer and produce the fiber [41].

As previously discussed, in nanofiber production from solution and melt polymer, voltage is the main factor affecting spinnability and fiber morphology. It is possible to reduce the diameter of the polymer fibers by applying higher electric field strengths by causing further stretching of the jet. Additionally, increasing the electric field strength is possible by increasing the supplied voltage or decreasing the needle tip-collector distance [42]. Qin et al. [43] investigated the effect of collector distance on fiber diameter, and the fiber diameter first decreased and then increased. The reason for this can be that the fiber stretching time is first prolonged in the electric field, and then the electrostatic pulling force weakens in the case of a large spinning distance. Although the surface tension of the low viscous polymer melt is almost close to that of the polymeric solution system, the viscosity of the polymer melt is higher than the viscosity of the polymer solution; therefore, more charge is required for melt electrospinning to initiate the jet than the solution electrospinning voltage [44]. In addition, because of the high viscosity and the rapid heat loss of the jet, larger fiber diameters are obtained in melt electrospinning. For instance, Sarwar et al. [45] investigated the effects of applied voltage, distance, and flow rate on fiber morphology while producing melt and solution from PEBA polymers. They showed that increasing voltage decreased fiber diameter from 7 to 5 μm. In both types of spinning techniques, the effect of process parameters on fiber diameter was found that similar results. The fibers’ minimum average diameters obtained by melt electrospinning were larger than solution electrospinning fibers. Additionally, Hao et al. studied the influence of temperature and voltage on the diameter of the melt-spinning polypropylene fibers. They found that with the 70–80 kV spinning voltage, they obtained good quality fibers with smooth surfaces and diameters smaller than 10 microns [23]. Morikawa et al. [46] showed that they could produce fibers with a diameter much closer to the fibers produced by solution electrospinning by developing a wire-based method based on the variation of the electrostatic fields around the spinneret instead of the needle-based method often used in melt electrospinning. In some cases, sub-micrometric fibers can be obtained because of the branching of larger fibers during the spinning of molten polymers. Because the jet may not solidify immediately after being ejected, it is subjected to stronger field forces as it moves towards the electric field. As a result, if the molecular weight of the polymer is low enough, side jets may form, leading to fibers with a smaller diameter than the molten jet [47].

The absence of solvents in melt spinning makes it a safer, greener, and completely automated method to produce fibers for various applications in the case of all the polymers, easily processed under controlled temperatures but requiring the use of highly toxic solvents to be dissolved [41]. However, it has complex pieces of equipment, ends up with a larger fiber diameter, and deficiency of suitable materials are a few problems.

### 3.2. Fibers from Polymer Solution

This method is based on fabricating an electrospun mesh from the polymer dissolved in a moderately volatile solvent, delivered to a spinneret, and exposed to a voltage potential (between the spinneret and a collecting surface) [48]. Any lower or higher applied voltage will result in beaded morphologies and inhibit polymer jet initiation. As the applied voltage rises above a critical value, the nanofiber diameter initially shrinks and increases after a certain point. This initial reduction in fiber diameter is due to the increase in voltage with increasing charges in the jet repelling each other and a high jet stretch. It is mainly reported that, with the increased applied voltage, the instability and stretching of the polymer jet also increase and generally lead to smaller fiber diameters [49]. Shao and co-workers investigated pullulan-CMC nanofibers modulated through process parameters (i.e., Polymer solution concentration, applied voltage, and feed rate) as a fruit packaging material. They showed that increasing the applied voltage from 19 to 21 kV and the feed rate from 0.36 to 0.6 mL/h leads to a reduction in mean fiber diameter from 0.187 to 0.092 µm [50]. In a previous study of the same group, the effect of applied voltage on the morphology of PVDF nanofiber mats was investigated, and the average fiber diameter decreased from 630 nm to 284 nm by increasing the voltage from 9 kV to 15 kV [51].

In a study, Herrero-Herrero and co-workers [52] investigated PLA/PCL fibers diameter as drug delivery systems and obtained under- and above-micron-sized fibers with the influence of majorly solvents ratio (chloroform/methanol and dichloromethane/dimethylformamide) and voltage. They showed that as the voltage increases, the diameter of the fibers decreases because of the stretching in the polymer jet associated with the charge repulsion.

In a different study, Nasouri and co-workers [53] aimed to model and optimize electrospun PAN nanofiber diameter using response surface methodology. They confirmed with the RSM analysis that polymer concentration and applied voltage were the main factors affecting the average nanofiber diameters. An increase in the applied voltage beyond the critical value will result in the formation of beads or beaded nanofibers. Increases in the diameter and formation of beads or beaded nanofibers with an increase in the applied voltage are attributed to the decrease in the size of the Taylor cone and the increase in the jet velocity for the same flow rate [54].

The preparation process for producing membranes with electrospinning from polymeric solutions frequently involves using unfriendly solvents for the environment and humans. In addition, final product electrospun fibers may contain traces of solvent after production. Generally, in this method, a polymer is dissolved in volatile solvents, namely trifluoroethanol (TFE), dimethylformamide (DMF), or dichloromethane (DCM) [55]. Considering the environmental risks and ecological damage they cause, the green production process in electrospinning is becoming widespread to produce biomaterials based on environmentally friendly resources. Accordingly, using green solvents with slow volatility may contribute to forming a fibrous network with a minimal effect on health and the environment. Recently, several studies have focused on using aqueous polymer solutions for electrospinning applications [56,57,58]. Alternatively, biologically benign solvents, such as formic acid or acetic acid, can be used alone or in combination as green solvent candidates. For instance, a recent study provided a comparative study on the fabrication of nanofibers from a PLGA and PCL solution in DMF: DCM and acetic acid, observing no significant difference in fiber morphologies and diameters in terms of using acetic acid [55].

A disadvantage of the green fabrication process is that the fibers present low physical stability, so a crosslinking strategy is required. In this case, green agents (e.g., citric acid) can be used in the place of other toxic chemicals (glutaraldehyde) for fiber crosslinking [59]. This approach is promising for processing eco-friendly sources, thus concurring to limit the destructive impact of petroleum-based products and relevant benefits in terms of environmental protection. For this purpose, the electrospinning technique is an optimal way to process biopolymers from natural sources such as chitin, chitosan, and pectin—to functional nanofibrous membranes [58]. For instance, in one study, a bio-based blend was successfully electrospun by combining small amounts of PEO with chitin propionate and green solvents, ethanol, and water [60]. This way, the electrospinning process is environmentally and human-friendly, fulfilling all basic sustainability principles better than other techniques. This process is summarized in Table 1, where a comparative analysis of the benefits/limitations of processing techniques based on using a solution or polymer melt to produce bulk or micro/nanofibrous films was reported.

## 4. Blended and Composite Fibers towards Bio-Sustainability

A polymer blend is a single-phase substance obtained by mixing two or more polymers, generally without strong chemical bonds. Polymeric composites are a multi-phase, multi-component system that combines polymeric and/or non-polymeric materials to improve their native properties by creating physical/chemical bonds. Especially in recent years, interest in polymers obtained from renewable resources has increased; thus, natural polymers, synthetic polymers from natural monomers, and polymers from microbial fermentation are frequently investigated. Similar to other petroleum-based polymers, polymers from renewable resources are rarely used alone, and many of their properties can be enhanced through blending and composite formation [64]. Micro- and nano-scale polymeric fibers have many advantages, such as large surface area and high porosity for use in biomedical applications [65], environmental filtrations [3], and textile products [66].

### 4.1. Biomedical Applications

In recent years, materials based on multi-component systems have been frequently investigated for the fabrication of nanomaterials with advanced mechanical and chemical properties [67] for biomedical use. Polymers with two or more synthetic or natural additives are generally used to obtain better properties. This way, desired chemical properties such as biocompatibility and biodegradability can be obtained, especially for functional tissue scaffolding [68,69]. Electrospinning enables the formation of nanofibers from a wide variety of materials, for example, polysaccharides, proteins, composite metals, metal oxides, ceramics, and carbon-containing biomaterials. Natural polymers (collagen, elastin, hyaluronic acid, and fibrinogen) commonly found in the native tissue extracellular matrix or derivative components obtained from natural sources such as gelatin, chitosan, silk fibroin, or vegetable oils are able to facilitate cell attachment, growth, and differentiation. Moreover, they are biodegradable and biocompatible.

Due to environmental needs, the development of bio-based fibers is becoming an increasingly hot topic and makes them interesting materials for environmental applications [67]. Despite the desirable properties, utilizing only natural polymers shows low spinnability. Additionally, one of the main disadvantages is their fast degradation rates due to their hydrophilic character and, in some cases, their poor mechanical properties, especially in wet environments [64]. To increase the spinnability of natural polymers, combining different polymers to formulate an electrospinning solution is a considerable strategy. Additionally, it is possible to integrate the desirable properties of natural and synthetic polymers into a single platform and fabricate various scaffolds with adjusted structural and chemical properties, depending on the intended application.

Synthetic polymers, for example, PCL, PLLA, PGA, and PLGA, remain important candidates for scaffold production because they have high mechanical strength and are structurally stable, easing tailoring the degradation rate. However, they are limited, especially in biomedical applications, when utilized alone because they lack functional parts and need modifications. Aliphatic polyesters, such as PLA, are known for their biodegradability and susceptibility to hydrolytic degradation. They have attracted much attention among commercial polymers as they are produced from renewable resources, are compostable, and have high mechanical performance [64]. Natural materials are mainly utilized effectively to achieve the desired biological properties (biocompatibility and bioactivity) lacking in other synthetic polymers. However, they must be stabilized by cross-linking or other methods in order to yield a stable material that can maintain its integrity [70,71]. In this case, polymer blending can enable the combination of complementary properties of different synthetic and natural polymers or can be used as a one-step surface modification technique to alter the wettability of electrospun mats [72].

**Figure 3:**
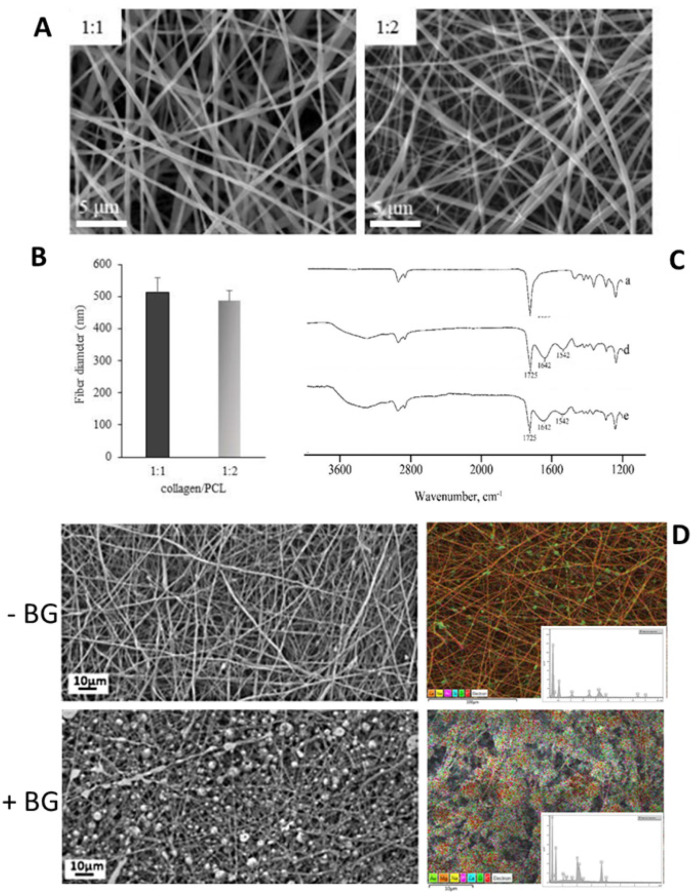
Composite (**A**–**C**) and blended (**D**) PCL-based fibers via green solvent electrospinning: (**A**) qualitative morphological analysis via SEM, (**B**) quantification of 1:1 and 1:2 PCL/Collagen blended nanofibers, and (**C**) FTIR analysis; (**D**) SEM and EDS spectra with element mapping of PCL/BG nanofibers [20,76]. (images from [20,76] with free license).

It is already known that nanofiber diameter, surface morphology, and pore size distribution of the mats obtained in electrospinning can be affected by electrospinning parameters. For instance, the type of polymer melt or solution, needle size, working distance, applied voltage, flow rate, and working environment could influence the final product’s properties [25]. The use of blends in the electrospinning system rather than a single polymer melt or solution system can affect the morphology and the chemical properties of the obtained mat. This effect is shown in a study to investigate the possibility of mixing PAN with different water-soluble polymers to change the morphology of the electrospun nanofibers. It was found that the nanofibers’ diameter was increased in the PAN/gelatin blend [73]. In another study, the fiber diameters of collagen–chitosan-TPU nanofiber scaffolds were found to be in the range of 256 ± 145 nm in the aligned fibers and lower than the fiber diameters in the random fibers, respectively. This is thought to be due to the stretching and weakening of the fibers with a high rotational speed. More importantly, many fine-diameter fibers (diameter ≤ 100 nm) were also seen in the scaffolds, which is explained by the positive charges carried by the chitosan in the blend. In other words, it is thought that the solution conductivity also increases with increasing loads, and the repulsive forces increase with the loads on the electrospinning jet, leading to further stretching of the fibers [74]. The choice of blend materials is also essential in electrospun membranes in tissue engineering applications because, in this way, fiber morphologies can be fabricated more suitable for tissue needs. In a study investigating the use of one- or two-component nanofiber channels in nerve tissue applications, the properties of nanofibers produced from the blend obtained by adding gelatin to the PCL polymer were compared with those produced using only PCL. The average (0.59 ± 0.15) μm diameter of the two-component fibrous channels obtained by adding gelatin solutions was in the submicron range. At the same time, PCL has been shown to consist of microfibers with an average diameter of (5.61 ± 0.8) μm [71,75]. Recently, it has been shown in the literature that toxic or hazardous solvents have been replaced with green solvents because of the effect of the chemicals on health and the environment. In addition, since solvents such as TFE are known to cause denaturation in the collagen structure, it has been shown that collagen and PCL-based nanofiber membranes can be successfully obtained by electrospinning by dissolving them in an aqueous solution of acetic acid (Figure 3) [76].

In a study aiming to examine the physicochemical properties of fibers consisting of collagen type I, collagen III, elastin, and polycaprolactone (PCL), when a 10% collagen and elastin blend is added to PCL, the fiber diameters obtained are thicker (310 nm), and where thick and flimsy fibers were observed this could be the reason of strong intermolecular bonds between proteins and forming bundle fibers and thus observing rounded surfaces instead of flat ones. When the polymer ratio is increased to 36%, thick and flimsy fibers are the majority (397 nm). When the effect of elastin and collagen was analyzed separately, the fiber diameters were obtained as 322 nm and 293 nm, respectively, in the case of 36% elastin and collagen added to PCL [77].

**Figure 4:**
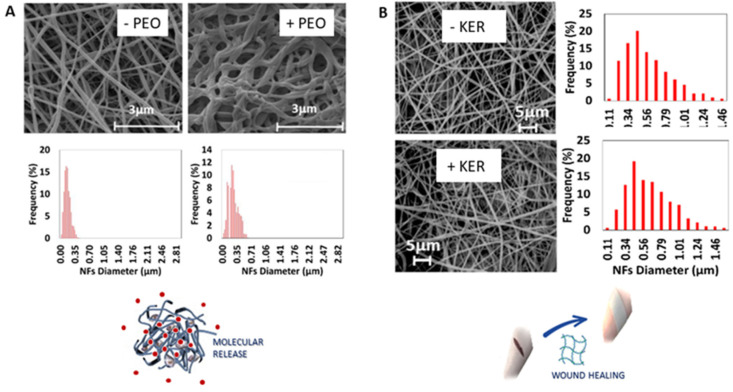
Keratin-based fibers processed via aqueous solvent electrospinning by adding hydrogel-like polymers: (**A**) PEO [78] and (**B**) PVA [79]: Fiber morphology via SEM and diameter distribution. (adapted from [78,79]).

More recently, the use of water solution to mix bioactive proteins with either synthetic or natural polymers was investigated to design more sustainable scaffolds able to improve cell interactions in vitro and in vivo. Ramirez et al. investigated the contribution of benign solvents in combination with additional polymers to address the properties of wool keratin nanofibers for drug delivery and wound healing applications (Figure 4). It is well known that solvent interactions can modify the spatial organization of macromolecular chains, playing an essential role in forming supramolecular structures and altering the electrospun fibers’ chemical and physical properties. They demonstrated that by blending keratin with hydrogel-like polymers, such as polyethylene oxide (PEO) (Figure 4a) [78] or polyvinyl alcohol (PVA) (Figure 4b) [79], the in vitro activity of cells can improve due to the local conditions of fluid and molecular transport that concur to form a better-recognized interface in terms of chemical stability and wettability features.

Alternatively, Vineis et al. [80] proposed the fabrication of protein-based nanofibers by dissolving a mix of three different proteins—gelatin, wool keratin, and sericin from silk fibroin—in an aqueous solution to validate their use for in vitro interaction studies. They investigated the combined effects of keratin and sericin on gelatin-based nanofibers’ chemical and physical properties, highlighting a correlation between physical/chemical interactions among proteins, i.e., a decrease in the hydrogen bonds of the N–H groups, and in vitro stability and cell response of nanofibers.

Polymer matrix reinforcement using fillers is commonly used in the production and processing of polymeric materials [64]. The fillers may geometries continuous (i.e., fibrous) or discontinuous geometries, such as short fibers, cubes, and blocks embedded in the polymers. They are used in a large volume concentration in polymers (>5 vol.%) [81]. Nanocomposite materials, in which the fillers are homogeneously dispersed in the polymer matrix and nanometric in sizes smaller than 100 nm (nanoparticles), differ from micro composites due to their unique properties and have great potential for advanced applications [82]. The properties of polymer micro/nanocomposites are affected by the nature, size, and shape of the polymer matrix and filler, the distribution of particles within the polymer matrix, and the thickness of the filler. Nanocomposites are one of the most researched areas in nanoscience, and currently, nano carbons, carbon nanotubes, graphene, nanodiamonds, and nanodots can be added to many electrospun functional polymers. It is widely used as a polymer matrix phase because it offers advantages in terms of processability compared to others, and it is known that nano carbons increase the functionality of electrospun polymeric fibers, enhance physical and mechanical properties and provide advantageous properties. For this purpose, it can be used to provide bio functionality as a scaffold in tissue engineering and sensors. Using this technique, composite electrospinning can form different polymers, such as PLA, PLGA, PCL, and PU, into different nanostructured materials. The choice of polymer for tissue engineering and biomedical applications is critical to achieving the target function [83,84,85].

It is known that the final product obtained with composite materials can also affect the nanofiber morphology. For example, it can be concluded that carbon nanotubes added to the polymeric matrix can cause an increase in solution viscosity, increasing fiber diameter. In one study, it was observed that electrospun nanofibers obtained from PAN polymer exhibited a smooth surface with a diameter of 200–400 nm. In contrast, no significant difference was observed in fiber diameters for PAN/CoFe_2_O_4_ fibers. The fibers obtained from the composite structure formed with CNTs had an increased diameter (400–700 nm). On the other hand, in PAN/CNT-CoFe_2_O_4_ fibers produced by electrospinning, the larger diameter approaching the microscale (approximately 700–1000 nm) suggests that it may be the result of a further increase in viscosity when CNTs and CoFe2O_4_ nanoparticles are added to the electrospinning solution [86].

In summary, it is possible to conclude that combining two or more materials with different physicochemical properties and phases—including polymers, ceramic, and/or metallic components—allows for fabricating composite materials with enhanced functional properties at the micro- and nanoscale. In this context, the definition of tailored setups can be relevant in producing composite and blending nanofibers from benign solvents. For instance, multi-needle electrospinning systems have a multi-needle setup to produce composite fibers from polymers that are not easily soluble in common solvents. Likewise, coaxial needles allow for the design of core–shell fibers in which a polymer solution (shell) and a compound solution (core) or two different polymer solutions (core–shell) can be assembled by controlling the spatial distribution of each phase, thus specifying the properties of the final device.

### 4.2. Environmental Applications: Contaminants Adsorption in Water and Their Sensing

Natural fibers are receiving growing interest due to their functionalization capabilities and relevant advantages in weight and fiber–matrix adhesion, especially in combination with polar matrix materials. They can be successfully used in waste management due to their biodegradability and much lower ash production after burning and post-processing. In this context, nano-based filler compounds, such as cellulose nanofibers, can also improve mechanical, physical, and gas-inhibitory properties. For instance, cellulose nanofibers were combined with starch to prepare nanocomposites acting as filters with high efficiency and accurate control of fiber mesh for particle separation [87]. Bates et al. [88] used electrospun chitosan–polyethylene oxide-oxidized cellulose composites to fabricate ultrafine membranes for wastewater applications with surface pore diameters less than 0.1 µm and uniform diameter distribution. Cellulose nanofibers are essential in the food industry as food packaging to match functional properties serving as gas or moisture absorbers, specific gas barriers, antimicrobial properties, and antioxidant activity [89].

In this frame, it is reported that the preparation of a novel cellulose-based nanofibrous membrane for separation of oil/water emulsions starts from an aqueous system coated with polydopamine (PDA), followed by grafting with polyethyleneimine (PEI). The as-prepared super-hydrophilic and underwater super-oleophobic membrane showed excellent separation performance for all different emulsions under broad pH range environments with separation efficiency above 99.1%. The membrane also exhibited good antifouling properties due to the development of a hydration layer underwater on the membrane surface. The reusability study indicated no noticeable decline in the permeation flux and separation efficiency even after 20 cycles [90].

Multicomponent membranes based on polyvinyl alcohol (PVA)/alginate/chitosan composite nanofibers containing nanomagnetic ZnO were studied for the removal of phenolic compounds from aqueous solutions. The composite nanofiber dosage and phenol solution temperature positively impact the phenol adsorption process. The thermodynamic parameters confirmed the endothermic and spontaneous nature of the phenol decontamination process onto the polymeric composite nanofibers. The fabricated composite nanofiber also revealed its capability for reuse after three cycles of regeneration [91].

**Figure 5:**
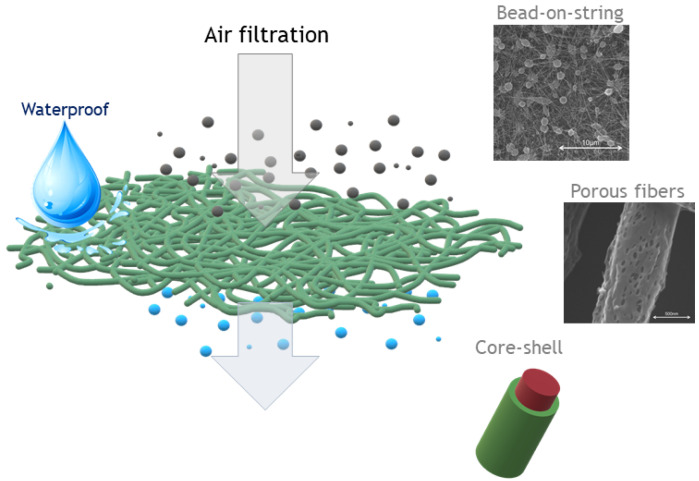
Scheme of a waterproof membrane suitable for air filtration/bio-filtration thanks to its structural features. Examples of structures and morphologies obtained by Electro Fluid Dynamic techniques: bead-on-string, porous and core/shell nanofibers.

In air filtration, electro fluid dynamic processes can be successfully used to design fibrous membranes with peculiar features in terms of morphology and composition and different structural order (Figure 5).

For instance, combining commercialized cellulose paper towels with zein electrospun fibers was realized for biodegradable composite filters, as the solvent selected was the well-known mixture of ethanol/water that led to both round and flat ribbon zein fibers. The results reveal that the filtration efficiency of the flat zein fiber air filter is 99% for PM0.3 removals and a low-pressure drop of 109 Pa, and it is much higher than that of the round zein fiber filer. These properties were also confirmed after 40 repeated filtration tests, and, more importantly, the developed filter was completely degraded after 42 h in cellulase solution [92].

**Figure 6:**
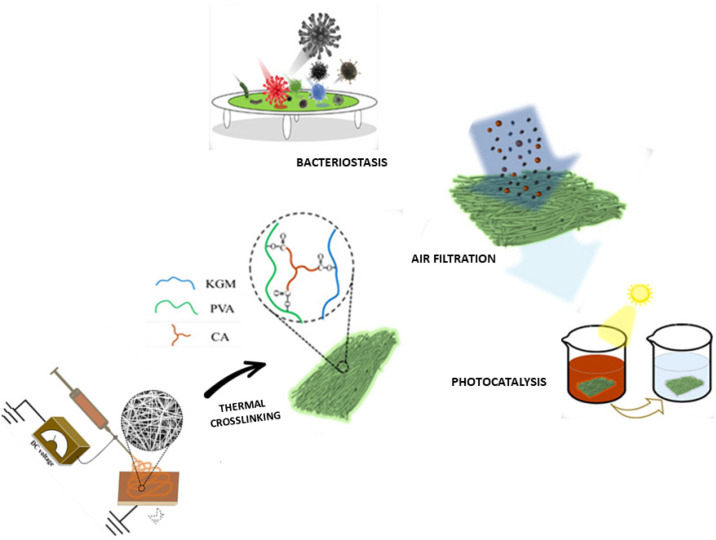
Schematic summary of processing routes based on EFDTs for the fabrication of active particles loaded systems via thermal crosslinking and examples of nanoparticle loaded membranes applications for environmental remediation, i.e., bacteriostasis, air filtration, photocatalysis (adapted from [93]).

For the same application, konjac glucomannan (KGM)/polyvinyl alcohol (PVA) electrospun membranes, loaded with ZnO nanoparticles, using water as a solvent and eco-friendly thermal cross-linking via citric acid and temperature were also realized (Figure 6). The obtained fibrous membranes show a filtration efficiency for ultrafine particles (300 nm) higher than 99.99%, superior to commercial HEPA filters [93].

Additionally, concerning petroleum-based polymers, the current trend is to realize a green electrospinning process, replacing toxic solvents for different applications.

Environmentally friendly PU nanofibrous waterproof and breathable membranes useful for broad-band applications such as in medical hygiene, wearable electronics, and outdoor clothing were realized via solvent emulsion electrospinning technique combined with heating treatment. A small amount of polyethylene oxide (PEO) was used as the template polymer, whereas trimethylolpropane tris (2-methyl-1-aziridine propionate) (TTMA) was selected as the crosslinking agent. Moreover, eco-friendly short perfluoroalkyl segments were introduced into the spinning emulsion to impart the hydrophobic character. The resultant water-processed nanofibrous membranes exhibit good waterproofness, impressive air permeability, outstanding water vapor transmission rate, and high elasticity [94].

Degradable and environmentally friendly polylactic acid (PLA) bead-on-string membranes to be used as air filtration masks were realized as well by green electrospinning using a mixture of relatively green solvents, ethyl acetate (EA)/N, N-dimethylformamide (DMF). Optimizing the solvent composition and polymer concentration made it possible to obtain the bead-on-sting morphology that led to an outstanding removal efficiency of over 98% for aerosol particles and an extremely low-pressure drop of 29.3 Pa. Furthermore, compared to commercial masks, the prepared PLA bead-on-string membrane shows higher filtration efficiency also in a real hazy environment, which results in a relatively safer PM index value [95].

As a membrane for seawater desalination, environmentally friendly alcohol-based polyamide mats were realized via one-step green solvent/non-solvent electrospinning by directly incorporating the fluorinated chemical into spinning solutions. The resultant polyamide nanofiber membranes exhibited high vapor permeation, good tensile strength, and elongation, thus permitting robust seawater desalination performance with remarkable salt rejection (99.97%) [96].

Lastly, petroleum-free polymers can be successfully used to fabricate membranes for an oil spill that is currently one of the major sources of water contamination. Generally, industrial or oil transport accidents can generate the uncontrolled release of crude oils into the oceans with relevant problems for environmental safety. This is imposed on developing cost-effective technology to reduce the environmental impacts caused by oil spills, overcoming the limitations of conventional strategies (i.e., mechanical recovery and chemical treatment) [97]. In this context, the electrospinning technique is a low-cost and highly versatile methodology that allows control over the fiber diameter. For instance, Zhu et al. reported the use of electrospun polyvinyl chloride/polystyrene nanofibers as sorbent for oil removal, with relevant benefits compared with commercial Polypropylene based nonwoven fabrics [98]. The peculiar sorption capacities of these fibers allow for the improvement of the performances of motor oil and diesel (i.e., 146 and 38 g/g, respectively) several times with respect to commercial sorbent fibers. Similar studies were conducted by Alnaqbi et al. that proposed the fabrication of recycled polystyrene micro-scaled fibers with a sorption capacity as high as 95 g/g of crude oil spills [99]. All of the previous works confirmed a relevant effect of process parameters (i.e., solvent type, voltage, flow rate, electrode distance, humidity degree) on the properties of fibrous sorbents to be used to minimize the damages related to crude oil spills in the sea. In this context, a step-by-step optimization of the process conditions is mandatory to improve the final performance of the fibrous sorbents for the definition of innovative and efficacious treatment of polluted seawater.

## 5. Monocomponent/Multicomponent Particles

### 5.1. Biomedical Applications

The production of micro- and nanosized particles represents an advantage for different applications in tissue engineering and drug delivery systems. For instance, microparticles can transport cells and display a longer retention time of molecules, while nanoparticles can easily penetrate barriers (i.e., brain–blood barriers) and cellular membranes [100,101]. In recent years, many studies have focused on the fabrication of polymer-based devices by EFDTs, particularly to encapsulate biomolecules to be used in drug delivery systems (antimicrobial and anticancer agents), theranostics, and tissue engineering [102].

EFDTs were originally used to fabricate particles to encapsulate hydrophobic and hydrophilic drugs for their attitude to high encapsulation efficiency and control of size in the range of micro- and nanoscale. For instance, cisplatin, an anticancer agent, was encapsulated into spherical and smooth PLGA nanoparticles via ES; Different formulations and process parameters, i.e., voltage and flow rates, were tested to optimize the final shape and size of nanoparticles. Further studies were performed to investigate the drug release, characterized by an initial burst release followed by a sustained release. For the higher content of cisplatin (10 wt.% with respect to PLA), the smallest burst release was recorded [103]. The initial burst release was attributed to the drug amount onto the particle surface, while the sustained part was mainly related to the peculiar properties of the polymer matrix (i.e., swelling, diffusion, degradation rate) [104]. PCL and PLGA microparticles have been fabricated to investigate in vitro the delivery of anticancer drugs. The release of paclitaxel was faster for loaded-PLGA than loaded-PCL microparticles in more than 30-day in vitro release [105]. The higher retention of drugs in PCL materials is related to its hydrophobic and slow degradation rate. For particular applications such as orthopedic grafts, the slow rate of degradation of PCL can be an advantage. For instance, melatonin has been encapsulated into PCL microparticles to inhibit osteoclastic activity and support bone remodeling [106].

**Figure 7:**
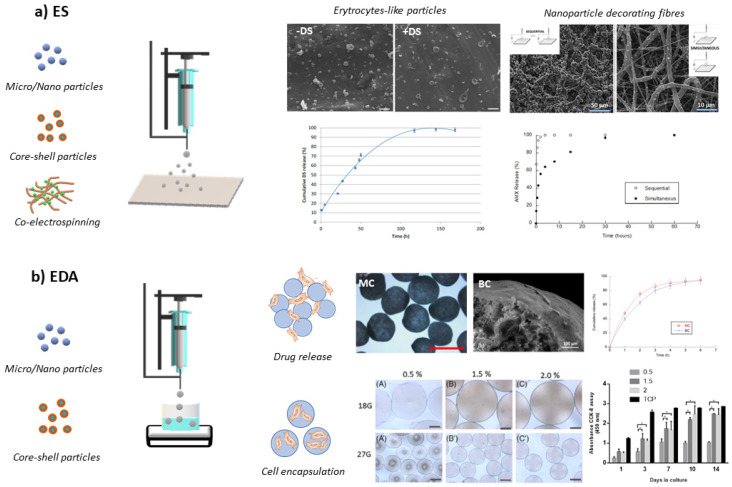
EFDTs for the fabrication of micro- and nano-devices. (**a**) electrospraying (ES): diclofenac-loaded (DS) erythrocyte-like nanoparticles—effect of DS on particle morphology and release profile (adapted from [110]); nanoparticle decorated fibers via co-electrospinning (i.e., simultaneous or sequential mode) for the development of fibrous platforms with different release of antibiotics from chitosan nanoparticles (adapted from [111]). (**b**) Electrodynamic technique (EDA): drug-loaded cellulose carriers with different compositions—i.e., mono-component (MC) or bi-component (BC) and effects on morphology and release profiles (adapted from [112]; alginate microgels for cell carrier/encapsulation—effect of polymer concentration on morphology and in vitro cell response (adapted from [113]).

Recently, the increased interest in using sustainable materials has also led to the use of materials from waste biomass and marine origin for biomedical applications [107]. Among them, chitosan is a non-toxic and biodegradable polymer that has become mostly used for the fabrication via ES of nanovectors loaded with different drugs (i.e., antibiotics [100], anti-inflammatory [108], and chemotherapeutics [109]). Moreover, other natural polymers, for example, alginate, chitosan, cellulose, collagen, gelatin, and hyaluronic acid, are currently being investigated, alone or blended in solution, to form mono- or multi-component particles (Figure 7).

Traditionally, an accredited approach involved the modification of natural and synthetic polymers to improve their chemical and physical properties. For instance, cellulose was grafted with PCL (cell-g-PCL) for the obtention of an amphiphilic polymer to design smart nanoparticles via ES for drug delivery applications using as drug model sodium diclofenac (DS) [110]. In this case, nanoparticles with an erythrocyte-like shape were obtained due to the intercalation of DS molecules between hydrophobic PCL branches grafted along the cellulose backbone, inducing an alteration of the entanglement density at the equilibrium. It was demonstrated that this interaction generates an initial burst release followed by a sustained release related to the cellulose phase and PCL, respectively.

More recently, different strategies were explored to fabricate multi-component particles via ES with increasing structural complexity in order to refine the drug release mechanisms for therapeutic applications. For example, multi-component PCL/silk fibroin particles loaded with anti-inflammatory and osteogenic factors were fabricated via monoaxial electrospraying [114]. The incorporation of silk fibroin overcomes the lack of surface functional groups and hydrophobicity of PCL, improving cell adhesion and growth. Moreover, the additional bioactivity conferred by dexamethasone and ascorbic acid induced the osteogenic differentiation of human adipose-derived stem cells and mineralization for bone tissue engineering. Gelatin methacryloyl (GelMA) microparticles were used as carriers of cells and growth factors for the treatment of degenerative disc disease Gelatin methacryloyl (GelMA) [115]. However, they present some drawbacks for oral administration related to limited targeting ability, short retention time in the gastrointestinal (GI) tract, and low bioavailability [116].

In order to better protect the active compounds, more complex experimental setups were optimized for the fabrication of drug carriers with core–shell architectures. For instance, cellulose acetate and chitosan have encapsulated and protected ketoprofen lysinate (KL) in the acid microenvironment [117]. In this case, the presence of the chitosan shell as a protective layer enables starting a progressive dissolution in the acid microenvironment, which eventually exposes the KL-cellulose core that retained the drug at gastric pH.

Additionally, one of the main issues in drug delivery is overcoming side effects due to inadequate drug concentration at the site of interest of systemic administration. In this regard, hierarchically structured composite systems can be fabricated for the local delivery of drugs [118,119]. For instance, it is preferred to avoid the formation of biofilms after chirurgical treatments in the periodontal pocket. Accordingly, the fabrication of amoxicillin-loaded chitosan nanoparticles entrapped into PCL nanofibrous networks was optimized by innovative processing routes based on a sequential or simultaneous deposition. A faster release of antibiotics was observed when the multi-component systems were prepared by sequential mode because the nanoparticles are especially dislocated onto the surface, with respect to simultaneous one, involving a more efficient entrapment of particles among the fibers, with major benefits in terms of release control and in vitro antibacterial response, for potential use in periodontal disease treatments [111]. A similar approach was followed to fabricate multi-component scaffolds of curcumin-loaded PLA particles into PCL/gelatin fibers for skin regeneration [120].

An alternative strategy involves using EFDTs to fabricate microgels, namely hydrogels at a micrometric size scale, which are suitable for drug and cell delivery. Recently, new experimental setups have been customized to process biopolymer solutions to fabricate different kinds of microgels. In particular, low-cost biopolymers from renewable resources, such as polysaccharides, are receiving particular attention due to their tunable properties (i.e., water adsorption, mechanical properties) that EFDTs processes can finely adjust for the fabrication of different devices [121].

For instance, alginates were widely processed via EHDA to produce mono-component microgels for cell carriers [122]. Alginate solution was atomized directly into a bath containing a crosslinking agent (i.e., BaCl_2_, CaCl_2_, FeCl_3_) [123] to fix size and shape. Further post-treatments are generally required to protect the encapsulated molecules or to functionalize the surface for cell adhesion. The fabrication of alginate microgels loaded with caffeine can be mentioned as a model for low molecular compound encapsulation. In this case, microgels were coated with chitosan to enhance caffeine retention [124].

As for a 3D in vitro model for cells, recent approaches have moved towards developing 3D culture into microgels with tailored properties to provide chemical and mechanical signals similar to the microenvironment of cells. In this regard, alginate microgels’ composition and process conditions were recently optimized to deeply investigate in vitro interaction with human mesenchymal stem cells (hMSCs) [113]. In order to include more efficient bioactive signals (i.e., adhesive or proliferative cues), mono-component microgels can be modified by adding proteins or growth factors to form multicomponent devices able to stimulate specific cell activities [125]. Chitosan was also proposed in combination with alginate to modulate the mechanical stiffness of the matrix to support and stimulate neuronal cultures [126]. Bio-inspired particles made of poly-D-lysine/alginate were used to encapsulate MSCs in order to protect them from immune cells, maintaining their immune-modulating functions suitable for systemic lupus erythematosus treatment [127].

### 5.2. Other Green Applications

**Figure 8:**
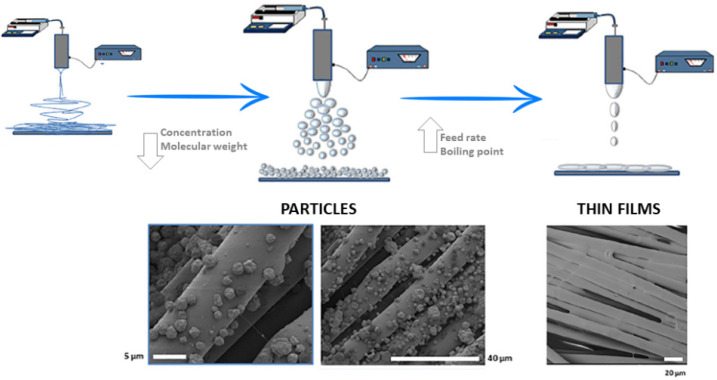
Scheme of experimental setup modifications of EFDTs for the fabrication of nanoparticles and thin films as sustainable coatings (images adapted from [128]). In comparison with the conventional setup used for the fabrication of electrospun fibers, the use of polymer solution with lower concentration/low molecular weight enables particles suitable for micro-nanostructured coatings. Likewise, a significant modification of fluid dynamic parameters (i.e., increase in flow rate) may also allow creation of a homogeneous and thin coating of porous substrates and textiles.

EFDTs with different configurations permit to attachment/decorate different substrates with micro/nanoparticles with controlled shapes/sizes for various applications such as separation membranes, antifouling sensors, active packaging, and innovative textiles (Figure 8).

In particular, ES is an easy and low-cost technique with the potential to create uniform multilayer thin coatings that can be scalable and manageable more quickly than a spin coating or dip coating [129]. Based on that, non-conventional EFDTs were also introduced—using electrostatic forces to polymer/composite solutions—to fabricate homogeneous coatings with tailored functionalities by a one-step process [128].

Different papers reported the use of electrosprayed interfacial polymerization (EIP) for fabricating polyamide-based membranes for nanofiltration. EIP simulates an additive manufacturing process with reciprocating scans of monomer spray; in this way, it is possible to control the thickness and facilitate the dissipation of reaction heat. Many strategies have been developed by tuning the surface structure, surface chemistry, and polyamide layer thickness to enhance separation performance. A polyamide composite nanofiltration membrane was realized starting from solutions of piperazine, benzenetricarbonyl trichloride, and Span 80 intercalation was used to coat a commercial membrane via EIP. The reduced thickness, surface roughness, and additional lamellar spacing nanochannels are responsible for good flux performance. Thanks to Span 80, high interfacial stability (polyamide layer and support layer), anti-backwashing ability, and 97.8% bisphenol A rejection are obtained [130].

ES can also be used to realize thickness-controllable selective layers of self-assembling materials onto commercial membranes via non-solvent-induced phase precipitation. Zwitterionic copolymers are able to self-assemble, thus creating domains that could be used to provide molecular-scale separation. The Zwitterionic and the non-solvent (isopropanol) solutions were deposited on the PAN400 ultrafiltration membrane to realize the copolymer layer. The results reveal that the ES printing technique enables the fabrication of a selective layer with a controllable thickness below 50 nm, high water permeability, and the complete rejection of chlorophyllin dye [131].

Another paper reports a similar procedure to realize ultrathin zwitterionic copolymer coatings onto an ion-selective membrane (ISM) used as the sensing layer of the ion-selective electrodes sensor (S-ISE). The copolymer and non-solvent solution were co-electrosprayed in order to induce copolymer precipitation. The coated membrane utilizes the microphase separation of the zwitterionic copolymer to form hydrophilic nanochannels acting as pores to mitigate biofouling without compromising the diffusion of primary ions (e.g., NH4+ for NH4+ S-ISE sensors) throughout the ISM matrix and augment the long-term accuracy and stability of the S-ISE sensors in wastewater [132].

A thin electrosprayed coating can also be realized on fabrics to obtain specific surface properties. A hydrophobic/superhydrophobic layer onto viscose fabric was obtained starting from a solution of Poly(tetrafluoroethylene) (PTFE), a synthetic fluoropolymer. First, the PTFE dispersion was electrosprayed, and then the sintering process was realized via a thermal treatment to activate the hydrophobicity and stabilize the PTFE-coated viscose fabric. The physical formation and adhesion of particles to the fibers were analyzed as a function of the sintering temperature. The fabric’s final properties were assessed in terms of self-cleaning property, anti-fouling property, and air and water vapor permeability. Coated fabrics increase their hydrophobicity by keeping breathability and water moisture transfer because of the difference between the PTFE-coated and uncoated sides [133].

ES has also been optimized to realize edible films and coatings acting as a reinforcement of the outer protective layer of foods (peel) and as a barrier to reduce the quality deterioration process. In particular, the feasibility of producing electrosprayed films with low and high-methoxyl pectin (LM and HM, respectively) added with recovered sunflower wax starting from water emulsion is reported. The results reveal that the addition of sunflower waxes to pectin films decreased molecular mobility and water molecule accessibility, reducing the rate of water vapor transmission. The films obtained with HM pectin resulted in more resistance, less flexibility, and greater stiffness than those from LM pectin [134].

Recently, A combination of electrospraying and spinning has been proposed to fabricate integrated Lithium-ion batteries (LIB). Thanks to the processes’ low cost, a wide range of raw materials, good process controllability, and high throughput, electrospinning and electrospraying technologies should be outstanding battery-manufacturing methods compared to spin coating, doctor blading, and paste coating. In particular, electrospinning was used to realize the polyacrylonitrile (PAN) separator, which was to prevent direct contact between the cathode and anode in case of a short battery circuit, to allow the fast transportation of Li+ ions and guarantee thermal stability and good mechanical properties. Then, the lithium iron phosphate (LiFePO4) cathode and commercial graphite anode are sprayed separately on both sides of the separator. The coated separator was then used to realize the LiFePO_4_||graphite full cell by electrolyte-injecting and packaging processes. The as-prepared full cell could light up a red light-emitting diode and provide high Coulombic efficiency of 97% and great cycling stability (capacity retention is 80.3%) [135].

## 6. Conclusions and Future Perspectives

In agreement with recent trends and political issues for environmental safety, sustainability is emerging as an essential criterion for developing innovative products suitable for biomedical and bio-environmental applications. For instance, a strong demand concerns the use of green technologies properly adapted to face the compelling need to ‘manufacture” biomaterials in combination with biological matter, i.e., cells, extracellular matrix (ECM), to produce even more complex living/not living products able to mimic the chemical and structural features of natural tissues.

Nowadays, substantial efforts are currently underway to synthesize new chemical and/or physical formulations to reach, within a few years, the definition of a new class of bio-sustainable materials that might combine the basic features of renewable natural resources and those of conventional biomaterials. In this view, significant attention should be focused on designing green processing routes with a lower energetic impact that should be progressively used by slowly replacing the traditional ones. Considering the different classes of biomaterials, from resorbable polymers such as polysaccharides and the proteins extracted from natural sources (i.e., plants and animals) to bioactive ones with solid interaction with living tissues can be successfully used in combination with fully sustainable and non-hazardous solvents, EFDTs are currently ready to face the challenge, allowing them to manipulate polymeric materials and their composites from renewable sources soluble in aqueous solutions, thus limiting the use of fossil-fuel-based products during product manufacturing. Meanwhile, new ideas to process via EFDs are under constant development due to their immense versatility and the high customization of the experimental setups. To date, more significant trends are mainly addressed to green manufacturing conditions involving the optimization of cross-linking reactions in a green environment to improve final devices’ structural and functional complexity.

In this perspective, a growing interest is emerging for the use of marine biopolymers to be efficiently used in the biomedical (i.e., cell scaffolds, bio-adhesives, molecular release, wound healing) and environmental application (i.e., water remediation). In this view, several works are suggesting the use of combinations of marine polymers (i.e., carrageenans, ulvans, fucoidans, laminarins)—usually characterized by low processability due to the absence of chains entanglements—with other biopolymers to obtain new formulations (i.e., blends, emulsions) with improved spinnability, suitable for the fabrication of nonwovens membranes by via co-electrospinning. This approach may contribute to overcoming some of the intrinsic limitations due to the poor mechanical properties and fast degradation of these materials in aqueous media. Meanwhile, the use of novel cross-linking methodologies based on the use of green compounds can offer the opportunity to modulate the mechanical properties in order to optimize polymer stability as a function of the required longevity in aqueous media, without significant decay of the main properties (i.e., hydrophilicity, stiffness). This approach could pave the way for the development of novel hybrid nanofibrous scaffolds with fascinating properties toward the more sustainable use of biomaterials.

## Figures and Tables

**Table 1:** Comparative analysis of fiber manufacturing via melt and solution processes.

Polymer State	HV	Size	Main Benefits	Limitations	Refs.
Melt	+	10 µm to 100 µm	-High structural control at the microscale; -No solvent use;	-Time-consuming to build. -Few polymers to test (thermal stability required) -High equipment costs -Hard to reach the nanoscale	[61]
Solution	+	<100 nm to 10 µm	-Highly customizable setup; -Low costs; -Suitable for many polymers; -Sub-micrometric scale	-High solvent volatility is required (few green solvent) -Low control of structural order (no direct writing)	[55]
Melt	-	50 µm to 500 µm	-High mass production -Weaving tech combination -Large-scale applicability	-High production cost -No diameter homogeneity -No nanometric scale	[62]
Solution	-	500 nm to 50 µm	-Thermally unstable polymer use; -Several configurations -Industrial use	-Solvent removal -Lack in morphological order (high defect occurrence)	[63]

## Data Availability

Not applicable.

## References

[1] Dai Z., Huang S. (2018). Functional Dynamics Inside Nano- or Microscale Bio-Hybrid Systems. Front. Chem..

[2] Tian F., Hosseinkhani H., Hosseinkhani M., Khademhosseini A., Yokoyama Y., Estrada G.G., Kobayashi H. (2008). Quantitative analysis of cell adhesion on aligned micro- and nanofibers. J. Biomed. Mater. Res. A.

[3] Yoon K., Hsiao B.S., Chu B. (2008). Functional nanofibers for environmental applications. J. Mater. Chem..

[4] Kim H.N., Jiao A., Hwang N.S., Kim M.S., Kim D.H., Suh K.Y. (2013). Nanotopography-guided tissue engineering and regenerative medicine. Adv. Drug Deliv. Rev..

[5] Alghoraibi I., Alomari S., Barhoum A., Bechelany M., Makhlouf A. (2018). Different Methods for Nanofiber Design and Fabrication. Handbook of Nanofibers.

[6] Guarino V., Ambrosio L. (2016). Electrofluidodynamics: Exploring a new toolbox to design biomaterials for tissue regeneration and degeneration. Nanomedicine.

[7] Haider S., Haider A. (2019). Electrohydrodynamic Processes and Their Affecting Parameters.

[8] Guarino V., Cirillo V., Altobelli R., Ambrosio L. (2015). Polymer-based platforms by electric field-assisted techniques for tissue engineering and cancer therapy. Expert Rev. Med. Devices.

[9] Aggarwal A., Sah M.K., Kasoju N., Ye H. (2021). Chapter Three—Electrospun materials as scaffolds in tissue engineering and regenerative medicine. Biomedical Applications of Electrospinning and Electrospraying.

[10] Del Bakhshayesh A.R., Babaie S., Niknafs B., Abedelahi A., Mehdipour A., Ghahremani-Nasab M. (2022). High efficiency biomimetic electrospun fibers for use in regenerative medicine and drug delivery: A review. Mater. Chem. Phys..

[11] Valachová K., el Meligy M.A., Šoltés L. (2022). Hyaluronic acid and chitosan-based electrospun wound dressings: Problems and solutions. Int. J. Biol. Macromol..

[12] Majumder S., Sagor M.M.H., Arafat M.T. (2022). Functional electrospun polymeric materials for bioelectronic devices: A review. Mater. Adv..

[13] Deng Y., Lu T., Cui J., Samal S.K., Xiong R., Huang C. (2021). Bio-based electrospun nanofiber as building blocks for a novel eco-friendly air filtration membrane: A review. Sep. Purif. Technol..

[14] Persano L., Camposeo A., Tekmen C., Pisignano D. (2013). Industrial Upscaling of Electrospinning and Applications of Polymer Nanofibers: A Review. Macromol. Mater. Eng..

[15] International Council for Harmonisation (2018). Impurities: Guideline for Residual Solvents Q3C (R7).

[16] Onoe H., Takeuchi S. (2015). Cell-laden microfibers for bottom-up tissue engineering. Drug Discov. Today.

[17] Ambhorkar P., Rakin R.H., Wang Z., Kumar H., Kim K. (2020). Biofabrication strategies for engineering heterogeneous artificial tissues. Addit. Manuf..

[18] Nie M., Takeuchi S. (2018). Bottom-up biofabrication using microfluidic techniques. Biofabrication.

[19] Shenoy S.L., Bates W.D., Frisch H.L., Wnek G.E. (2005). Role of chain entanglements on fiber formation during electrospinning of polymer solutions: Good solvent, non-specific polymer–polymer interaction limit. Polymer.

[20] Hao M.F., Liu Y., He X.T., Ding Y.M., Yang W.M. (2011). Factors Influencing Diameter of Polypropylene Fiber in Melt Electrospinning. Adv. Mater. Res..

[21] Bambole V., Yakhmi J.V., Grumezescu A.M. (2016). Chapter 14—Tissue engineering: Use of electrospinning technique for recreating physiological functions. Nanobiomaterials in Soft Tissue Engineering.

[22] Haider A., Haider S., Kang I.-K. (2018). A comprehensive review summarizing the effect of electrospinning parameters and potential applications of nanofibers in biomedical and biotechnology. Arab. J. Chem..

[23] Şener A.G., Altay A.S., Altay F. Effect of voltage on morphology of electrospun nanofibers. Proceedings of the 2011 7th International Conference on Electrical and Electronics Engineering (ELECO).

[24] Gade H., Nikam S., Chase G.G., Reneker D.H. (2021). Effect of electrospinning conditions on β-phase and surface charge potential of PVDF fibers. Polymer.

[25] Deitzel J.M., Kleinmeyer J., Harris D., Tan N.C.B. (2001). The effect of processing variables on the morphology of electrospun nanofibers and textiles. Polymer.

[26] Bagbi Y., Pandey A., Solanki P.R., Thomas S., Pasquini D., Leu S.-Y., Gopakumar D.A. (2019). Chapter 10—Electrospun Nanofibrous Filtration Membranes for Heavy Metals and Dye Removal. Nanoscale Materials in Water Purification.

[27] Edikresnha D., Suciati T., Khairurrijal K. (2021). Preliminary study of composite fibers polyvinylpyrrolidone/cellulose acetate loaded by garlic extract by means of electrospinning method. Mater. Today Proc..

[28] Unnithan A.R., Arathyram R.S., Kim C.S., Thomas S., Grohens Y., Ninan N. (2015). Chapter 3—Electrospinning of Polymers for Tissue Engineering. Nanotechnology Applications for Tissue Engineering.

[29] Liverani L., Boccaccini A.R. (2016). Versatile Production of Poly(Epsilon-Caprolactone) Fibers by Electrospinning Using Benign Solvents. Nanomaterials.

[30] Van der Schueren L., de Schoenmaker B., Kalaoglu Ö.I., de Clerck K. (2011). An alternative solvent system for the steady state electrospinning of polycaprolactone. Eur. Polym. J..

[31] Pires L.R., Guarino V., Oliveira M.J., Ribeiro C.C., Barbosa M.A., Ambrosio L., Pêgo A.P. (2016). Ibuprofen-loaded poly(trimethylene carbonate-co-ε-caprolactone) electrospun fibres for nerve regeneration. J. Tissue Eng. Regen. Med..

[32] Altobelli R., Guarino V., Ambrosio L. (2016). Micro- and nanocarriers by electrofludodynamic technologies for cell and molecular therapies. Process Biochem..

[33] Jaworek A. (2007). Micro- and nanoparticle production by electrospraying. Powder Technol..

[34] Guarino V., Khodir W.K.W.A., Ambrosio L. (2012). Biodegradable microparticles and nanoparticles by electrospraying techniques. J. Appl. Biomater. Funct. Mater..

[35] Smeets A., Clasen C., van den Mooter G. (2017). Electrospraying of polymer solutions: Study of formulation and process parameters. Eur. J. Pharm. Biopharm..

[36] Daly A.C., Riley L., Segura T., Burdick J.A. (2020). Hydrogel microparticles for biomedical applications. Nat. Rev. Mater..

[37] Guarino V., Altobelli R., Cirillo V., Cummaro A., Ambrosio L. (2015). Additive electrospraying: A route to process electrospun scaffolds for controlled molecular release. Polym. Adv. Technol..

[38] Enayati M., Chang M.W., Bragman F., Edirisinghe M., Stride E. (2011). Electrohydrodynamic preparation of particles, capsules and bubbles for biomedical engineering applications. Colloids Surf. A Physicochem. Eng. Asp..

[39] Guarino V., Ambrosio L., Bellini D. (2009). Process for the Preparation of Microspheres Comprising Semisynthetic. Polymers. Patent.

[40] Mayol L., Borzacchiello A., Guarino V., Serri C., Biondi M., Ambrosio L. (2014). Design of electrospayed non-spherical poly (L-lactide-co-glicolide) microdevices for sustained drug delivery. J. Mater. Sci. Mater. Med..

[41] Guarino V., D’Albore M., Altobelli R., Ambrosio L. (2016). Polymer Bioprocessing to Fabricate 3D Scaffolds for Tissue Engineering. Int. Polym. Process..

[42] Zhang L.-H., Duan X.P., Yan X., Yu M., Ning X., Zhao Y., Long Y.Z. (2016). Recent advances in melt electrospinning. RSC Adv..

[43] Qin C.-C., Duan X.P., Wang L., Zhang L.H., Yu M., Dong R.H., Long Y.Z. (2015). Melt electrospinning of poly(lactic acid) and polycaprolactone microfibers by using a hand-operated Wimshurst generator. Nanoscale.

[44] Bubakir M.M., Li H., Barhoum A., Yang W., Barhoum A., Bechelany M., Makhlouf A.H. (2018). Advances in Melt Electrospinning. Handbook of Nanofibers.

[45] Sarwar Z., Krugly E., Danilovas P.P., Ciuzas D., Kauneliene V., Martuzevicius D. (2019). Fabrication and characterization of PEBA fibers by melt and solution electrospinning. J. Mater. Res. Technol..

[46] Morikawa K., Green M., Naraghi M. (2018). A Novel Approach for Melt Electrospinning of Polymer Fibers. Procedia Manuf..

[47] Lyons J., Li C., Ko F. (2004). Melt-electrospinning part I: Processing parameters and geometric properties. Polymer.

[48] Goldstein A.S., Thayer P.S., Grumezescu A.M. (2016). Chapter 11—Fabrication of complex biomaterial scaffolds for soft tissue engineering by electrospinning. Nanobiomaterials in Soft Tissue Engineering.

[49] Pillay V., Dott C., Choonara Y.E., Tyagi C., Tomar L., Kumar P., Ndesendo V.M. (2013). A Review of the Effect of Processing Variables on the Fabrication of Electrospun Nanofibers for Drug Delivery Applications. J. Nanomater..

[50] Shao P., Niu B., Chen H., Sun P. (2018). Fabrication and characterization of tea polyphenols loaded pullulan-CMC electrospun nanofiber for fruit preservation. Int. J. Biol. Macromol..

[51] Shao H., Fang J., Wang H., Lin T. (2015). Effect of electrospinning parameters and polymer concentrations on mechanical-to-electrical energy conversion of randomly-oriented electrospun poly(vinylidene fluoride) nanofiber mats. RSC Adv..

[52] Herrero-Herrero M., Gómez-Tejedor J.A., Vallés-Lluch A. (2018). PLA/PCL electrospun membranes of tailored fibres diameter as drug delivery systems. Eur. Polym. J..

[53] Nasouri K., Bahrambeygi H., Rabbi A., Shoushtari A.M., Kaflou A. (2012). Modeling and optimization of electrospun PAN nanofiber diameter using response surface methodology and artificial neural networks. J. Appl. Polym. Sci..

[54] Deitzel J.M., Kleinmeyer J.D., Hirvonen J.K., Tan N.C.B. (2001). Controlled deposition of electrospun poly(ethylene oxide) fibers. Polymer.

[55] Mosher C.Z., Brudnicki P.A., Gong Z., Childs H.R., Lee S.W., Antrobus R.M., Lu H.H. (2021). Green electrospinning for biomaterials and biofabrication. Biofabrication.

[56] Lee S.Y., Jeong Y.J., Park W.H. (2022). Poly(vinyl alcohol) nanofibrous membranes via green electrospinning and tannin coating for selective removal of Pb(II) ion. Chemosphere.

[57] Tonda-Turo C., Ruini F., Ceresa C., Gentile P., Varela P., Ferreira A.M., Ciardelli G. (2018). Nanostructured scaffold with biomimetic and antibacterial properties for wound healing produced by ‘green electrospinning’. Colloids Surf. B Biointerfaces.

[58] Celebioglu A., Saporito A.F., Uyar T. (2022). Green Electrospinning of Chitosan/Pectin Nanofibrous Films by the Incorporation of Cyclodextrin/Curcumin Inclusion Complexes: pH-Responsive Release and Hydrogel Features. ACS Sustain. Chem. Eng..

[59] Zhu M., Hua D., Zhong M., Zhang L., Wang F., Gao B., Huang C. (2018). Antibacterial and Effective Air Filtration Membranes by ‘Green’ Electrospinning and Citric Acid Crosslinking. Colloid Interface Sci. Commun..

[60] Zhong T., Liu W., Liu H. (2021). Green electrospinning of chitin propionate to manufacture nanofiber mats. Carbohydr. Polym..

[61] Robinson T.M., Hutmacher D.W., Dalton P.D. (2019). The Next Frontier in Melt Electrospinning: Taming the Jet. Adv. Funct. Mater..

[62] Gajjar C.R., Stallrich J.W., Pasquinelli M.A., King M.W. (2021). Process–Property Relationships for Melt-Spun Poly(lactic acid) Yarn. ACS Omega.

[63] Xu Z., Wu M., Ye Q., Chen D., Liu K., Bai H. (2022). Spinning from Nature: Engineered Preparation and Application of High-Performance Bio-Based Fibers. Engineering.

[64] Yu L., Dean K., Li L. (2006). Polymer blends and composites from renewable resources. Prog. Polym. Sci..

[65] Ghajarieh A., Habibi S., Talebian A. (2021). Biomedical Applications of Nanofibers. Russ. J. Appl. Chem..

[66] Liu L., Xu W., Ding Y., Agarwal S., Greiner A., Duan G. (2020). A review of smart electrospun fibers toward textiles. Compos. Commun..

[67] Angel N., Li S., Yan F., Kong L. (2022). Recent advances in electrospinning of nanofibers from bio-based carbohydrate polymers and their applications. Trends Food Sci. Technol..

[68] Sionkowska A. (2021). Collagen blended with natural polymers: Recent advances and trends. Prog. Polym. Sci..

[69] Hanumantharao S.N., Rao S. (2019). Multi-Functional Electrospun Nanofibers from Polymer Blends for Scaffold Tissue Engineering. Fibers.

[70] Renkler N.Z., Ergene E., Gokyer S., Ozturk M.T., Huri P.Y., Tuzlakoglu K. (2021). Facile modification of polycaprolactone nanofibers with egg white protein. J. Mater. Sci. Mater. Med..

[71] Cirillo V., Clements B.A., Guarino V., Bushman J., Kohn J., Ambrosio L. (2014). A comparison of the performance of mono- and bi-component electrospun conduits in a rat sciatic model. Biomaterials.

[72] Guarino V., Cirillo V., Ambrosio L. (2016). Bicomponent electrospun scaffolds to design extracellular matrix tissue analogs. Expert Rev. Med. Devices.

[73] Wehlage D., Böttjer R., Grothe T., Ehrmann A. (2018). Electrospinning water-soluble/insoluble polymer blends. AIMS Mater. Sci..

[74] Huang C., Chen R., Ke Q., Morsi Y., Zhang K., Mo X. (2011). Electrospun collagen–chitosan–TPU nanofibrous scaffolds for tissue engineered tubular grafts. Colloids Surf. B Biointerfaces.

[75] Fasolino I., Guarino V., Cirillo V., Ambrosio L. (2017). 5-Azacytidine-mediated hMSC behavior on electrospun scaffolds for skeletal muscle regeneration. J. Biomed. Mater. Res. A.

[76] Miele D., Catenacci L., Rossi S., Sandri G., Sorrenti M., Terzi A., Bonferoni M.C. (2020). Collagen/PCL Nanofibers Electrospun in Green Solvent by DOE Assisted Process. An Insight into Collagen Contribution. Materials.

[77] Aguirre-Chagala Y.E., Altuzar V., León-Sarabia E., Tinoco-Magaña J.C., Yañez-Limón J.M., Mendoza-Barrera C. (2017). Physicochemical properties of polycaprolactone/collagen/elastin nanofibers fabricated by electrospinning. Mater. Sci. Eng. C.

[78] Ramirez D.O.S., Cruz-Maya I., Vineis C., Guarino V., Tonetti C., Varesano A. (2021). Wool Keratin-Based Nanofibres-In Vitro Validation. Bioengineering.

[79] Ramirez D.O.S., Cruz-Maya I., Vineis C., Tonetti C., Varesano A., Guarino V. (2021). Design of Asymmetric Nanofibers-Membranes Based on Polyvinyl Alcohol and Wool-Keratin for Wound Healing Applications. J. Funct. Biomater..

[80] Vineis C., Maya I.C., Mowafi S., Varesano A., Ramírez D.S., Abou Taleb M., El-Sayed H. (2021). Synergistic effect of sericin and keratin in gelatin based nanofibers for in vitro applications. Int. J. Biol. Macromol..

[81] Xanthos M. (2010). Functional Fillers for Plastics.

[82] Pleşa I., Noţingher P.V., Schlögl S., Sumereder C., Muhr M. (2016). Properties of Polymer Composites Used in High-Voltage Applications. Polymers.

[83] Ghosal K., Agatemor C., Špitálsky Z., Thomas S., Kny E. (2019). Electrospinning tissue engineering and wound dressing scaffolds from polymer-titanium dioxide nanocomposites. Chem. Eng. J..

[84] Lee J.K.Y., Chen N., Peng S., Li L., Tian L., Thakor N., Ramakrishna S. (2018). Polymer-based composites by electrospinning: Preparation & functionalization with nanocarbons. Prog. Polym. Sci..

[85] Watanabe K., Maeda T., Hotta A. (2018). Uniformly dispersed polymeric nanofiber composites by electrospinning: Poly(vinyl alcohol) nanofibers/polydimethylsiloxane composites. Compos. Sci. Technol..

[86] Ni Q.Q., Jin X.D., Xia H., Liu F., Zhang D. (2014). 7—Electrospinning, processing and characterization of polymer-based nano-composite fibers. Advances in Filament Yarn Spinning of Textiles and Polymers.

[87] Kallu S., Kowalski R.J., Ganjyal G.M. (2017). Impacts of Cellulose Fiber Particle Size and Starch Type on Expansion During Extrusion Processing. J. Food Sci..

[88] Bates I.I.C., Loranger É., Mathew A.P., Chabot B. (2021). Cellulose reinforced electrospun chitosan nanofibers bio-based composite sorbent for water treatment applications. Cellulose.

[89] Vasile C., Baican M. (2021). Progresses in Food Packaging, Food Quality, and Safety-Controlled-Release Antioxidant and/or Antimicrobial Packaging. Molecules.

[90] Wang J., Wang L. (2020). Superhydrophilic and underwater superoleophobic nanofibrous membrane for separation of oil/water emulsions. J. Mater. Res..

[91] Elkady M., Salama E., Amer W.A., Ebeid E.-Z.M., Ayad M.M., Shokry H. (2020). Novel eco-friendly electrospun nanomagnetic zinc oxide hybridized PVA/alginate/chitosan nanofibers for enhanced phenol decontamination. Environ. Sci. Pollut. Res. Int..

[92] Hu J., Xiong Z., Liu Y., Lin J. (2022). A biodegradable composite filter made from electrospun zein fibers underlaid on the cellulose paper towel. Int. J. Biol. Macromol..

[93] Lv D., Wang R., Tang G., Mou Z., Lei J., Han J., Huang C. (2019). Ecofriendly Electrospun Membranes Loaded with Visible-Light-Responding Nanoparticles for Multifunctional Usages: Highly Efficient Air Filtration, Dye Scavenging, and Bactericidal Activity. ACS Appl. Mater. Interfaces.

[94] Zhou W., Gong X., Li Y., Si Y., Zhang S., Yu J., Ding B. (2022). Environmentally friendly waterborne polyurethane nanofibrous membranes by emulsion electrospinning for waterproof and breathable textiles. Chem. Eng. J..

[95] Han W., Rao D., Gao H., Yang X., Fan H., Li C., Meng H. (2022). Green-solvent-processable biodegradable poly(lactic acid) nanofibrous membranes with bead-on-string structure for effective air filtration: ‘Kill two birds with one stone’. Nano Energy.

[96] Zhou W., Zhang X., Gong X., Ding M., Yu J., Zhang S., Ding B. (2022). Environmentally Friendly Polyamide Nanofiber Membranes with Interconnective Amphiphobic Channels for Seawater Desalination. ACS Appl. Mater. Interfaces.

[97] Adebajo M.O., Frost R.L., Kloprogge J.T., Carmody O., Kokot S. (2003). Porous Materials for Oil Spill Cleanup: A Review of Synthesis and Absorbing Properties. J. Porous Mater..

[98] Zhu H., Qiu S., Jiang W., Wu D., Zhang C. (2011). Evaluation of Electrospun Polyvinyl Chloride/Polystyrene Fibers as Sorbent Materials for Oil Spill Cleanup. Environ. Sci. Technol..

[99] Alnaqbi M.A., Greish Y.E., Mohsin M.A., Elumalai E.J., al Blooshi A. (2016). Morphological variations of micro-nanofibrous sorbents prepared by electrospinning and their effects on the sorption of crude oil. J. Environ. Chem. Eng..

[100] Oliveira M.B., Mano J.F. (2011). Polymer-based microparticles in tissue engineering and regenerative medicine. Biotechnol. Prog..

[101] Barua S., Mitragotri S. (2014). Challenges associated with penetration of nanoparticles across cell and tissue barriers: A review of current status and future prospects. Nano Today.

[102] Bock N., Dargaville T.R., Woodruff M.A. (2012). Electrospraying of polymers with therapeutic molecules: State of the art. Prog. Polym. Sci..

[103] Parhizkar M., Reardon P.J., Knowles J.C., Browning R.J., Stride E., Barbara P.R., Edirisinghe M. (2016). Electrohydrodynamic encapsulation of cisplatin in poly (lactic-co-glycolic acid) nanoparticles for controlled drug delivery. Nanomed. Nanotechnol. Biol. Med..

[104] Kamaly N., Yameen B., Wu J., Farokhzad O.C. (2016). Degradable Controlled-Release Polymers and Polymeric Nanoparticles: Mechanisms of Controlling Drug Release. Chem. Rev..

[105] Xie J., Marijnissen J.C.M., Wang C.-H. (2006). Microparticles developed by electrohydrodynamic atomization for the local delivery of anticancer drug to treat C6 glioma in vitro. Biomaterials.

[106] Gurler E.B., Ergul N.M., Ozbek B., Ekren N., Oktar F.N., Haskoylu M.E., Gunduz O. (2019). Encapsulated melatonin in polycaprolactone (PCL) microparticles as a promising graft material. Mater. Sci. Eng. C.

[107] Soares R.M.D., Siqueira N.M., Prabhakaram M.P., Ramakrishna S. (2018). Electrospinning and electrospray of bio-based and natural polymers for biomaterials development. Mater. Sci. Eng. C Mater. Biol. Appl..

[108] Guarino V., Altobelli R., Ambrosio L. (2016). Chitosan Microgels and Nanoparticles via Electrofluidodynamic Techniques for Biomedical Applications. Gels.

[109] Başpinar Y., Akbaba H., Bayraktar O. (2019). Encapsulation of paclitaxel in electrosprayed chitosan nanoparticles. J. Res. Pharm..

[110] Guarino V., Altobelli R., Caputo T., Ambrosio L., Caserta S., Calcagnile P., Demitri C. (2019). Mono- and Bi-Phasic Cellulose Acetate Micro-Vectors for Anti-Inflammatory Drug Delivery. Pharmaceutics.

[111] Zuppolini S., Maya I.C., Diodato L., Guarino V., Borriello A., Ambrosio L. (2020). Self-associating cellulose-graft-poly(ε-caprolactone) to design nanoparticles for drug release. Mater. Sci. Eng. C.

[112] Gandhimathi C. (2015). Controlled Release of Dexamethasone in PCL/Silk Fibroin/Ascorbic Acid Nanoparticles for the Initiation of Adipose Derived Stem Cells into Osteogenesis. J. Drug Metab. Toxicol..

[113] Xu H., Sun M., Wang C., Xia K., Xiao S., Wang Y., Chen L. (2020). Growth differentiation factor-5–gelatin methacryloyl injectable microspheres laden with adipose-derived stem cells for repair of disc degeneration. Biofabrication.

[114] Hasan M.N., Shahriar S.M.S., Mondal J., Nurunnabi M., Lee Y., Nurunnabi M. (2021). Chapter Six—Bioinspired and biomimetic materials for oral drug delivery. Bioinspired and Biomimetic Materials for Drug Delivery.

[115] Guarino V., Caputo T., Calcagnile P., Altobelli R., Demitri C., Ambrosio L. (2018). Core/shell cellulose-based microspheres for oral administration of Ketoprofen Lysinate. J. Biomed. Mater. Res. Part B Appl. Biomater..

[116] Yang G., Li X., He Y., Ma J., Ni G., Zhou S. (2018). From nano to micro to macro: Electrospun hierarchically structured polymeric fibers for biomedical applications. Prog. Polym. Sci..

[117] Khodir W.W.A., Guarino V., Alvarez-Perez M., Cafiero C., Ambrosio L. (2013). Trapping tetracycline-loaded nanoparticles into polycaprolactone fiber networks for periodontal regeneration therapy. J. Bioact. Compat. Polym..

[118] Guarino V., Cruz-Maya I., Altobelli R., Khodir W.A., Ambrosio L., Pèrez M.A.A., Flores A.A. (2017). Electrospun polycaprolactone nanofibres decorated by drug loaded chitosan nano-reservoirs for antibacterial treatments. Nanotechnology.

[119] Saadipour M., Karkhaneh A., Nazarpak M.H. (2022). An investigation into curcumin release from PLA particles loaded in PCL-GELATIN fibers for skin application. Int. J. Polym. Mater. Polym. Biomater..

[120] Muthukumaran P., Babu P.S., Karthikeyan S., Kamaraj M., Aravind J. (2021). Tailored natural polymers: A useful eco-friendly sustainable tool for the mitigation of emerging pollutants: A review. Int. J. Environ. Sci. Technol..

[121] Xu M., Qin M., Cheng Y., Niu X., Kong J., Zhang X., Wang H. (2021). Alginate microgels as delivery vehicles for cell-based therapies in tissue engineering and regenerative medicine. Carbohydr. Polym..

[122] Bajpai S.K., Sharma S. (2004). Investigation of swelling/degradation behaviour of alginate beads crosslinked with Ca^2+^ and Ba^2+^ ions. React. Funct. Polym..

[123] Nikoo A.M., Kadkhodaee R., Ghorani B., Razzaq H., Tucker N. (2018). Electrospray-assisted encapsulation of caffeine in alginate microhydrogels. Int. J. Biol. Macromol..

[124] Cruz-Maya I., Altobelli R., Marrese M., Guarino V. (2021). Design of alginate based micro-gels via electro fluid dynamics to construct microphysiological cell culture systems. Polym. Adv. Technol..

[125] Murphy A.R., Laslett A., O’Brien C.M., Cameron N.R. (2017). Scaffolds for 3D in vitro culture of neural lineage cells. Acta Biomater..

[126] Tedesco M.T., Di Lisa D., Massobrio P., Colistra N., Pesce M., Catelani T., Pastorino L. (2018). Soft chitosan microbeads scaffold for 3D functional neuronal networks. Biomaterials.

[127] Nie M., Chen G., Zhao C., Gan J., Alip M., Zhao Y., Sun L. (2021). Bio-inspired adhesive porous particles with human MSCs encapsulation for systemic lupus erythematosus treatment. Bioact. Mater..

[128] Guarino V., Varesano A., Focarete M.L., Gualandi C., Ramakrishna S. (2018). Electrospinning Technology for Filtering Membranes Fabrication. Filtering Media by Electrospinning: Next Generation Membranes for Separation Applications.

[129] De Falco F., Guarino V., Gentile G., Cocca M., Ambrogi V., Ambrosio L., Avella M. (2019). Design of functional textile coatings via non-conventional electrofluidodynamic processes. J. Colloid Interface Sci..

[130] Yang S., Wang J., Fang L., Lin H., Liu F., Tang C.Y. (2020). Electrosprayed polyamide nanofiltration membrane with intercalated structure for controllable structure manipulation and enhanced separation performance. J. Membr. Sci..

[131] Qian X., Ravindran T., Lounder S.J., Asatekin A., McCutcheon J.R. (2021). Printing zwitterionic self-assembled thin film composite membranes: Tuning thickness leads to remarkable permeability for nanofiltration. J. Membr. Sci..

[132] Huang Y., Qian X., Wang X., Wang T., Lounder S.J., Ravindran T., Li B. (2022). Electrospraying Zwitterionic Copolymers as an Effective Biofouling Control for Accurate and Continuous Monitoring of Wastewater Dynamics in a Real-Time and Long-Term Manner. Environ. Sci. Technol..

[133] Yang K., Peng Q., Venkataraman M., Novotna J., Karpiskova J., Mullerova J., Militky J. (2022). Hydrophobicity, water moisture transfer and breathability of PTFE-coated viscose fabrics prepared by electrospraying technology and sintering process. Prog. Org. Coat..

[134] Chalapud M.C., Baümler E.R., Carelli A.A., Salgado-Cruz MD L.P., Morales-Sánchez E., Rentería-Ortega M., Calderón-Domínguez G. (2022). Pectin Films with Recovered Sunflower Waxes Produced by Electrospraying. Membranes.

[135] Hu Z., Hao J., Shen D., Gao C., Liu Z., Zhao J., Lu B. (2022). Electro-spraying/spinning: A novel battery manufacturing technology. Green Energy Environ..

